# Influence of the Type of Cement and the Addition of an Air-Entraining Agent on the Effectiveness of Concrete Cover in the Protection of Reinforcement against Corrosion

**DOI:** 10.3390/ma14164657

**Published:** 2021-08-18

**Authors:** Wioletta Raczkiewicz, Peter Koteš, Petr Konečný

**Affiliations:** 1 Department of Concrete Construction and Geotechnics, Faculty of Civil Engineering and Architecture, Kielce University of Technology, Al. 1000 lecia PP 7, 25-314 Kielce, Poland; 2 Department of Structures and Bridges, Faculty of Civil Engineering, University of Žilina, Univerzitná 8215/1, 010 26 Žilina, Slovakia; peter.kotes@uniza.sk; 3 Department of Structural Mechanics, Faculty of Civil Engineering, VŠB—Technical University of Ostrava, Ludvika Podéště 1875, 70800 Ostrava, Czech Republic; petr.konecny@vsb.cz

**Keywords:** reinforcement corrosion, chloride ions, freezing and thawing cycles, cement type, air-entraining agent, galvanostatic pulse technique

## Abstract

The concrete cover is the basic protection of the reinforcement against the influence of external factors that may lead to its corrosion. Its effectiveness depends mainly on the composition of the concrete mix, including the cement used. Depending on external environmental factors that may aggressively affect the structure, various types of cements and concrete admixtures are recommended. The paper presents the results of tests that allow us to assess the effect of the type of cement used and the air-entraining agent on the effectiveness of the concrete cover as a layer protecting the reinforcement against corrosion. In order to initiate the corrosion process, the reinforced concrete specimens were subjected to cycles of freezing and thawing in a sodium chloride solution. The degree of advancement of the corrosion process was investigated using the electrochemical galvanostatic pulse technique. Additionally, the microstructure of specimens taken from the cover was observed under a scanning electron microscope. The research has shown that in the situation of simultaneous action of chloride ions and freezing cycles, in order to effectively protect the reinforcement against corrosion, the application of both blast-furnace slag cement and an air-entraining agent performed the best.

## 1. Introduction

Reinforcement corrosion is the main reason for reducing the durability of reinforced concrete structures [1,2,3,4,5]. For this reason, research is constantly being carried out to maximize the protection of steel bars against corrosion or to assess the possibility of replacing black steel with more corrosion-resistant reinforcement. The effectiveness of reinforcement made of galvanized steel is tested [6], including in an aggressive chloride environment [7,8]. Experimental tests also concern specimens and elements of the structures in which steel bars are replaced with a non-metallic reinforcement made of glass fiber (GFRP) [9,10], carbon (CFRP) [11], basalt [12] or even aramid (AFRP) [13]. It was also confirmed that the use of randomly dispersed polymer fibres (FRC) has a positive effect on reducing the corrosion of the reinforcement in concrete [14,15]. The conducted tests prove the effectiveness of the use of alternative forms of reinforcement in order to reduce corrosion in concrete elements. However, at the same time, the authors point to other problems, e.g., related to the limited adhesion of rods to concrete [7,11], a different (rapid, uncontrolled) damage mechanism of elements in which such reinforcement was used [11,13], the difficult workability of the concrete mix with the addition of fibres [14,15], high performance requirements [7,8] or the high cost of such reinforcement.

For the above reasons, despite the increasing use of non-metallic reinforcement, the use of black steel is still the most popular in concrete structures. It is also important from the point of view of the existing elements of the structures. Therefore, solutions are still being sought that allow the most effective protection of this type of reinforcement against the influence of negative environmental factors that may lead to the development of corrosion on the surface of reinforcing bars [16,17,18,19]. Such protection should be provided by the concrete cover, which, on the one hand (due to the highly alkaline pH of concrete, pH ≈ 12.5–13.5) contributes to the formation of a passive layer on the surface of the bars, and on the other hand (due to the appropriate thickness and tightness) reduces or slows down the rate of penetration into the concrete structure of aggressive substances that may initiate corrosion on the reinforcement. Depending on the future operating conditions of the structure (exposure class [20]), the appropriate cover thickness and the appropriate composition of the concrete mix are selected to make its effectiveness as effective as possible.

For structures where carbonation is the main risk, Portland cement is used for concrete. In concretes with Portland cement, the carbonation process is slower than in concretes with other types of cement, due to the higher content of calcium hydroxide, which is formed in the hydration of calcium silicates from Portland clinker [4,21,22].

However, in concrete structures that may be exposed to chemical aggression, it is recommended to use the blast-furnace slag cements or cement with supplementary cementitious materials for concrete [23] and some authors recommend cements containing granular blast furnace slag in particular [24,25,26]. As a result of the use of blast-furnace slag cement, which during hydration produces a greater amount of the C-S-H gel phase with a reduced content of C_3_A compared to Portland cement [4], the concrete setting and hardening processes are slower, and the concrete structure is more compact and tight, with a lower content of capillary pores. Due to the lower heat of hydration, shrinkage strains in concrete with blast-furnace slag cement are smaller and occur more slowly [4,24,25,26]. This reduces the penetration of aggressive substances into the concrete structure, which is especially important in the case of extremely aggressive, pitting chloride corrosion. Diffusion of chloride ions, which are the strongest depasivators of steel, occurs faster than CO_2_, diffusion, and the corrosion of the reinforcement is severe [27,28,29,30,31].

Damage to the concrete cover and, consequently, an increase in the probability of corrosion of the reinforcement, may occur as a result of frost and cycled freezing and thawing of the liquid in the concrete pores. The internal stresses that arises can lead to numerous microcracks in the lagging, which reduces its tightness [1,2,16,32,33,34]. In order to protect the structural elements in such cases, it is recommended to add air-entraining agents to the concrete mix [33,34,35]. By adding an air-entraining agent, the concrete forms air bubbles or closed pores that are not completely filled with water, which take excess water from the capillary pores during ice crystallization. In addition, the air voids formed are a place of deposition of some chemical reaction products, thanks to which no stresses that could damage the concrete structure arise [4,32].

Despite many publications on the protection of reinforcement in reinforced concrete structures against aggressive environmental factors (that may lead to the initiation and development of reinforcement corrosion) only a few concern research on the simultaneous effect of chloride ions and frost and are usually based on studies of real structures [16,31]. In turn, laboratory tests often relate to independent tests of material, concrete and steel. Therefore, it is worth undertaking laboratory tests that allow us to assess the effect of selected components of a concrete mix, i.e., the type of cement and the addition of an air-entraining agent on effectiveness of concrete in protecting the reinforcement against chloride corrosion and frost. The paper presents the results of such tests.

The tests were performed using the non-destructive electrochemical galvanostatic pulse technique [36,37,38,39]. This method was developed and used in the measurements of the corrosion of reinforcement in concrete as a more advanced technique [40,41,42,43] in comparison to the commonly used method of measuring the half-cell potential [44]. The half-cell potential method sometimes led to a misinterpretation of the results, especially in the structures placed in a wet and anaerobic environment. The galvanostatic pulse technique made it possible to estimate the size of the corrosion probability in the tested specimens and to assess the corrosion activity of the tested reinforcement in a relatively simple and quick way.

## 2. Materials and Methods

### 2.1. Test Specimens

The tests were performed on reinforced concrete specimens. The concrete mix was prepared using alternatively two types of cements: Portland cement (CEM I 42.5 N-MSR/NA, Lafarge Cement S.A., Małogoszcz, Poland) or blast furnace slag cement (CEM III/A 42.5 N-LH/HSR/NA, Lafarge Cement S.A., Małogoszcz, Poland). Moreover, in both groups of specimens made with different cements, an air-entraining agent was used in half of them. In total, 24 specimens were used and divided into 4 groups, with 6 specimens in each group:

(a) C_I_a specimens—specimens made of concrete with Portland cement and the addition of an air-entraining agent;
(b) C_I_n specimens—specimens made of concrete with Portland cement, without the addition of an air-entraining agent;
(c) C_III_a specimens—specimens made of concrete with blast-furnace slag cement and the addition of an air-entraining agent;
(d) C_III_n specimens—specimens made of concrete with blast-furnace slag cement, without the addition of an air-entraining agent.

The components of a concrete mix for 1 m^3^ are included in Table 1. The concrete additives—plasticizer and an air-entraining agent—were dosed in a percentage of the cement volume.

**Figure 1:**
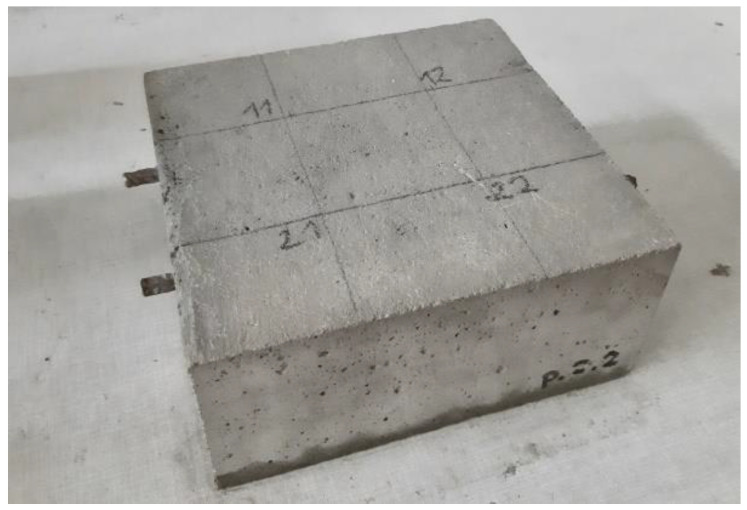
A photo of one of the research specimens.

The specimens were 210 mm × 228 mm × 100 mm. In each specimen, there were placed two parallel ribbed bars φ 8 mm diameter, made of BST 500 steel, placed 70 mm apart from the side edges of the specimen. The assumed cover was 25 mm (Figure 1).

The specimens were made under identical conditions in the laboratory hall. The specimens were removed from the moulds the day after concreting and cured in water for a period of 14 days. After taking them out of the water, the specimens were stored in a laboratory hall at a temperature of 20 ± 2 °C and a relative humidity of 50 ± 5%. In order to initiate the corrosion process on the reinforcement, the samples were immersed in a 3% sodium chloride solution (3% NaCl) and simultaneously 120 cycles of freezing and thawing in a climatic chamber in temperature cycles from −18 to +18 °C.

The measurements of estimating the size of the corrosion probability and the corrosive activity of the reinforcement included:

(a) stage I—reference measurements made 90 days after concreting the specimens, but before the freeze–thaw cycles;
(b) stage II—measurements made 250 days after concreting the specimens, after 120 cycles of freezing and thawing.

The measurements were made using the non-destructive electrochemical galvanostatic pulse method. In addition, using a scanning electron microscope (SEM), the microstructure of selected specimens from each group was observed (C_I_a, C_I_n, C_III_a, C_III_n) after the freeze–thaw cycles.

### 2.2. Galvanostatic Pulse Technique

The non-destructive electrochemical galvanostatic pulse method is described in detail among others in [36,39,45,46]. The basis of this method is the fact that the corrosion process of reinforcement in concrete is an electrochemical process in which a steel reinforcing bar is an electrode and the alkaline liquid filling the pores of the concrete is an electrolyte. A local anode and cathode formed on the surface of the bar generate the flow of electric charge through electricity, while the liquid filling the pores of the concrete is the carrier of ions. The non-destructive electrochemical test methods developed on this basis allow to indirectly assess the advancement of steel corrosion in concrete on the basis of the measurement of certain electrical quantities (which indicate the ongoing corrosion process of the reinforcement) [19,36,38] and referring them to the limit criteria values (Table 2). These measurements concern: the steady-state potential of the reinforcement (half-cell potential/stationary potential, E_st_), the resistivity of the concrete cover (Ɵ) measured on the concrete surface and the corrosion current density (i_cor_). In order to measure the corrosion current density, it is necessary to polarize the reinforcing bar (i.e., to disturb the dynamic equilibrium of the corrosive system that prevails on an electrode—rebar, immersed in an electrolyte—concrete with pores filled with liquid). Such a disturbance can be caused by an electrical impulse. Depending on the method of generating the pulse, different polarization methods have been developed. In the galvanostatic pulse method, the disturbance is generated by a current of a certain intensity.

The measurements of the reinforcement steady-state potential and the concrete cover resistivity are called basic measurements. It should be noted that the values of these parameters are only estimates of the probability of the reinforcement corrosion risk in the investigated areas and the measurement results are not direct and are indications only, e.g., concrete cover resistivity depends significantly on the concrete composition on the top corrosion process [47].

The so-called advanced measurements, which rely on additional measurements of the corrosion current density and make it possible to estimate the corrosive activity of the reinforcement and to predict its rate over time, are much more reliable.

**Figure 2:**
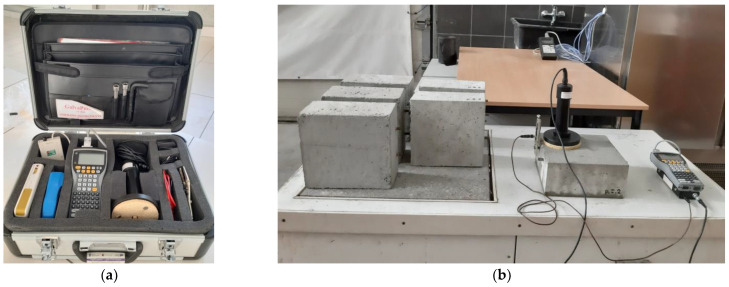
A photo of the test stand: (**a**) elements of the GP-5000 GalvaPulse^TM^ set, (**b**) scheme of connection of the set with the reinforcement in the tested specimen.

The article presents the results obtained on the basis of advanced measurements. The measurements were made using the GP-5000 GalvaPulse™ (Force Technology, Brøndby, Denmark) set [36]. This device allows the simultaneous measurement of three electrical quantities: half-cell reinforcement potential (E_st_), concrete cover resistivity (Θ) and the corrosion current density (i_cor_). The main elements of the set include the control and recording device (PSION minicomputer, GI Germann Instruments, Copenhagen, Denmark), silver-chloride reference electrode (Ag/AgCl) and calibration device. The elements of the set (including additional equipment) fit into a medium-sized suitcase (Figure 2a). The half-cell potential is measured to an accuracy of ±5 mV with the Ag/AgCl electrode. The electrical resistance is estimated to be measured with an accuracy of ±5%. The advantage of using the GalvaPulse™ set (compared to devices used in half-cell potential measurements) is a relatively uncomplicated operation and short measurement time—usually no more than thirty seconds at point.

Measurements of the relevant parameters were performed at two measurement points for each rebar in each specimen. The measurement points were placed along the rebar position line at equal intervals every 70 mm (Figure 2b). Thus, for each specimen group, the results were obtained from 24 measurement points (4 measurement points for each of the six specimens in a given group).

The obtained results (presented in the diagrams in Figures 3–14 and in Tables 3–5) were compared to the criteria for corrosion risk evaluation presented in Table 2 [36].

Depending on the obtained values of the reinforcement half-cell potential and concrete cover resistivity, it was possible to conclude about the value of the corrosion probability of the reinforcement in the studied area, and on the basis of the value of the corrosion current density, it was possible to estimate the corrosion activity of the reinforcement. However, it should be noted that of the three parameters mentioned, the most reliable measurement is the corrosion current density, because this parameter is a direct measure of the corrosive activity of steel and allows us to estimate the rate of corrosion development on the tested rebar in relation to the current environmental conditions. The other two parameters, i.e., the half-cell potential of the reinforcement and the resistivity of the concrete cover, allow for an indirect and approximate estimation of the probability of corrosion in the area under investigation (especially since the manufacturer of GalvaPulse did not specify for which parameters of the concrete mix and for which environmental conditions are appropriate the criteria presented in Table 2). The results obtained from the measurements of these two parameters are thus less reliable, but they complete the analysis based on the measurement of the corrosion current density.

## 3. Results and Discussion

### 3.1. The Corrosion Current Density

**Figure 3:**
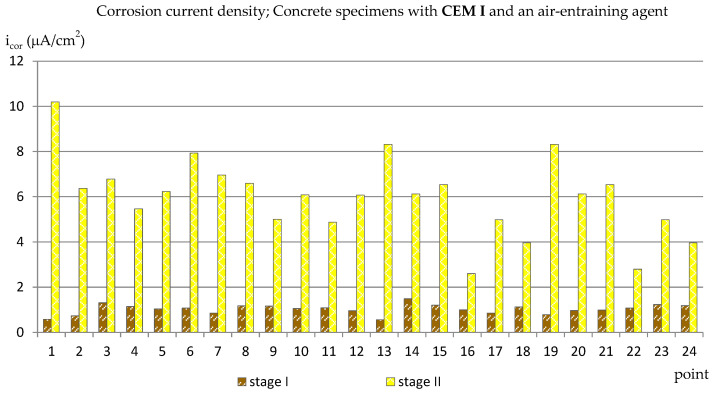
Corrosion current density measured on the reinforcement in C_I_a specimens.

**Figure 4:**
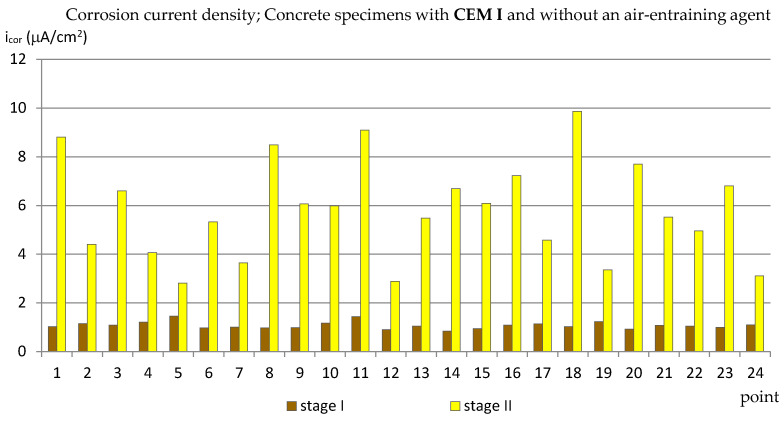
Corrosion current density measured on the reinforcement in C_I_n specimens.

**Figure 5:**
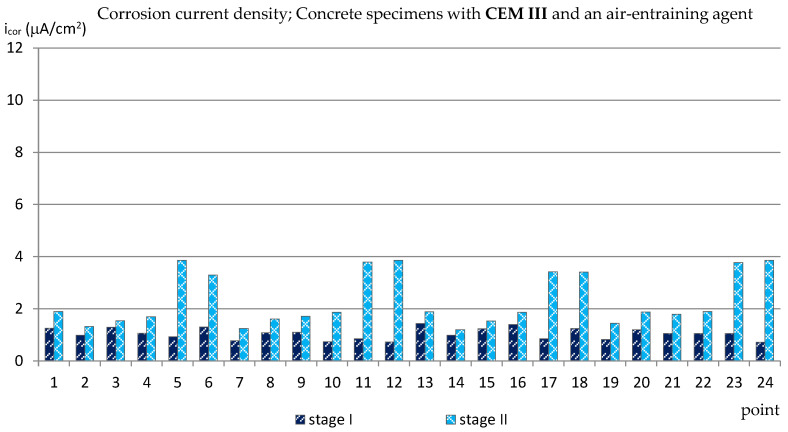
Corrosion current density measured on the reinforcement in C_III_a specimens.

**Figure 6:**
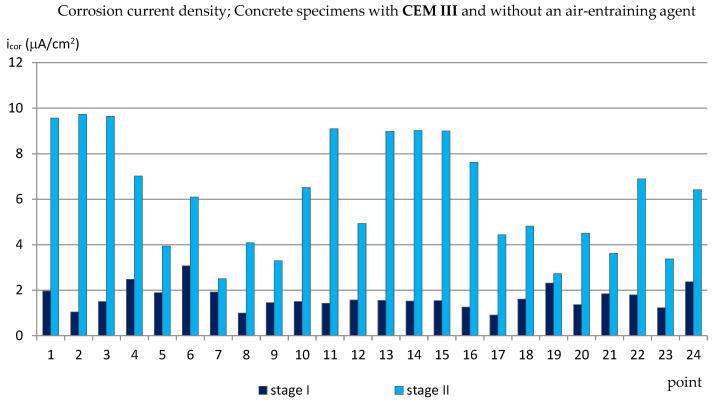
Corrosion current density measured on the reinforcement in C_III_n specimens.

The results of the corrosion current density measurements are presented in the form of graphs in the Figure 3, Figure 4, Figure 5 and Figure 6, where the first two graphs concern specimens in which Portland cement was used (CEM_I), and the next two concern specimens with blast-furnace slag cement (CEM III). In both cases, respectively, the results are shown first for specimens with an air-entraining agent (C_I_a, C_III_a), and then for specimens without an air-entraining agent (C_I_n, C_III_n). Additionally, Table 3 shows the extreme values of the corrosion current density measured before (reference measurement) and after the freezing cycles in all groups of specimens.

The reference measurements (made before the specimens were subjected to freezing and thawing cycles) showed that the values of the corrosion current density measured at all the designated points in the specimens from groups C_I_a, C_I_n and C_III_a and in most points in the samples from the C_III_n group (Figure 3, Figure 4, Figure 5 and Figure 6) were in the range of i_cor_ = 0.56 ÷ 1.48 μA/cm^2^, which, in relation to the given criterion, testified to irrelevant corrosion activity of reinforcement. Only in four points in specimens C_III_n did the corrosion current density exceeded i_cor_ = 2.0 μA/cm^2^ (maximum i_cor_ = 3.07 μA/cm^2^) indicating low corrosion activity. Such results may be related to the delayed setting time and the formation of a passive layer on reinforcement in concretes where the blast-furnace slag cement was used.

The results of the measurements performed after the completed 120 cycles of freezing and thawing the specimens in a 3% sodium chloride solution were already quite varied depending on what cement was used and whether or not an air-entraining agent was added. In samples C_I_a (Figure 3), in most points, the corrosion current density values exceeded i_cor_ = 5.0 μA/cm^2^ (most of them oscillated around i_cor_ ≈ 6.0 μA/cm^2^; in three points i_cor_ ≈ 8 μA/cm^2^, and in one point i_cor_ = 10.19 μA/cm^2^) which indicated moderate corrosive activity. Only in four points did the corrosion current not density exceed i_cor_ = 4 μA/cm^2^.

In the second group of Portland cement specimens, C_I_n (Figure 4), in which no air-entraining agent was used, the limit values of the results were similar to those obtained in specimens C_I_a (from i_cor_ = 2.82 μA/cm^2^ to i_cor_ = 9.87 μA/cm^2^), but the results were more dispersive and there were more results with lower values than for the C_I_a specimens. The comparison of the results in both groups of specimens with a Portland cement shows that the use of an air-entraining agent does not reduce the corrosion activity of the reinforcement caused by the action of chlorides and frost. Moreover, in the C_I_n specimens (without the air-entraining agent), most of the results indicated lower corrosion activity of the reinforcement than in the C_I_a specimens. On the other hand, the addition of an air-entraining agent resulted in a more uniform corrosion activity of the reinforcement in the studied areas.

The analysis of the results of the corrosion current density measurements carried out for specimens with blast-furnace slag cement showed that in C_III_a specimens, in which an air-entraining agent was used, the results of measurements of this parameter made after 120 cycles of freezing and thawing specimens in a 3% sodium chloride solution increased only slightly in relation to the reference measurements (Figure 5). At most measuring points, the corrosion current density did not exceed i_cor_ = 2.0 μA/cm^2^, which pointed to irrelevant corrosion activity of reinforcement. Only in eight points was the value of this parameter higher and equal to i_cor_ ≈ 3.3 ÷ 3.86 μA/cm^2^, which (despite a very aggressive corrosive environment) meant a low corrosion activity. 

On the other hand, the values of the corrosion current density measured in the C_III_n specimens (without the air entraining agent) turned out to be similar to the values obtained in the C_I_n specimens and amounted from i_cor_ = 2.51 μA/cm^2^ to i_cor_ = 9.72 μA/cm^2^ (Figure 6). This proves that the use of only blast-furnace slag cement, but without the simultaneous addition of an air-entraining agent, does not reduce the corrosion of the reinforcement caused by the simultaneous action of chloride ions and frost.

### 3.2. The Half-Cell Potential of Reinforcement

**Figure 7:**
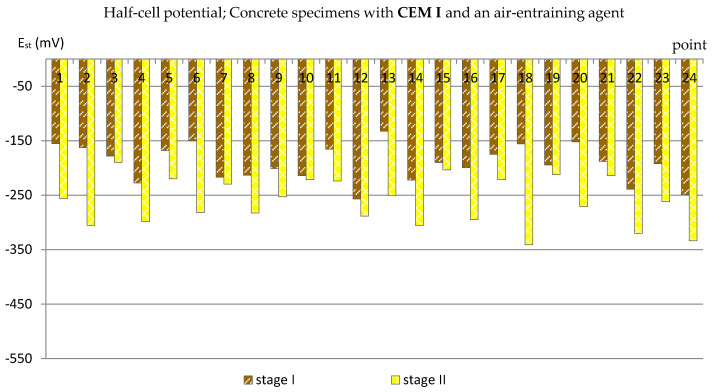
Half-cell potential of reinforcement measured in C_I_a specimens.

**Figure 8:**
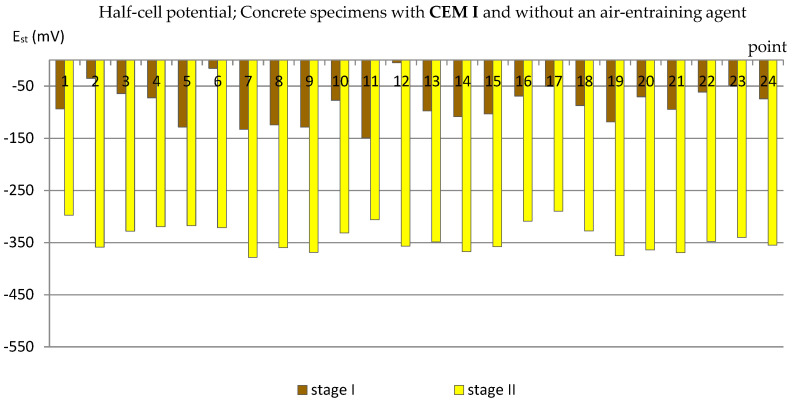
Half-cell potential of reinforcement measured in C_I_n specimens.

**Figure 9:**
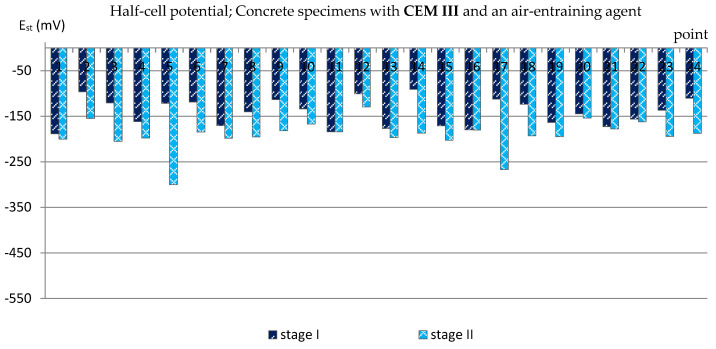
Half-cell potential of reinforcement measured in C_III_a specimens.

**Figure 10:**
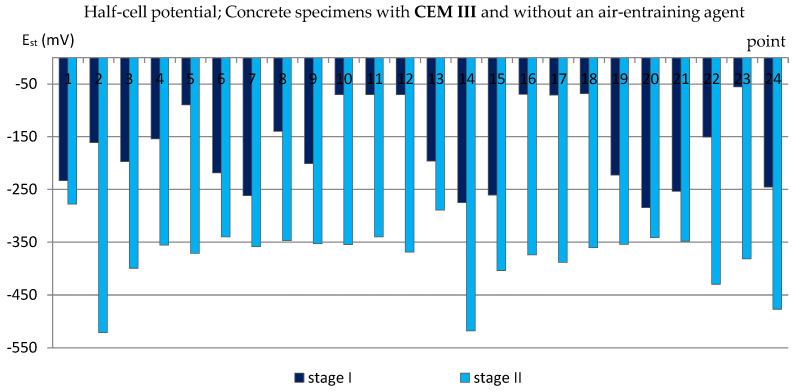
Half-cell potential of reinforcement measured in C_III_n specimens.

A similar analysis was performed based on the results of measurements of the reinforcement half-cell potential (Figure 7, Figure 8, Figure 9 and Figure 10; Table 4). However, it should be remembered that this parameter is no longer as reliable as the corrosion current density and only allows to estimate the probability of corrosion of the reinforcement in a small area under examination. Table 4 presents the extreme values of the reinforcement half-cell potential obtained from measurements of all types of specimens before and after the cycles of freezing and thawing them in a 3% NaCl solution.

Observing the results of measurements of the reinforcement half-cell potential obtained from the tests of all groups of specimens (C_I_a, C_I_n, C_III_a, C_III_n) performed after the specimens freezing and thawing cycles in a 3% NaCl solution, a clear influence of the air entraining agent on the reduction of the probability of rebars corrosion can be noticed. In C_I_a specimens (with Portland cement and an air-entraining agent), in most of the measurement points, the values of the reinforcement half-cell potential were E_st_ = (−189 ÷ −300) mV, and were only five points lower than E_st_ = −300 mV (Figure 7), which made it possible to estimate the probability of corrosion of the reinforcement at the level of 5% ÷ 50% (according to Table 2). 

Meanwhile, the results obtained from the measurements on C_I_n specimens (with Portland cement but without an air-entraining agent) were much lower—at 22 measurement points they exceeded E_st_ = −300 mV, including 12 points lower than E_st_ = −350 mV (Figure 8), which indicated a 95% probability of corrosion of the reinforcement in these areas. 

Even more clearly, the influence of the air-entraining agent could be observed in the specimens with blast-furnace slag cement. The values of the reinforcement half-cell potential obtained from the measurements performed after the cycles of freezing and thawing in specimens in which an air-entraining agent was used (C_III_a) in almost all measurement points did not exceed E_st_ = −200 mV (Figure 9), which allowed us to estimate the probability of corrosion of the reinforcement at the level of 5%. Only at two points did the half-cell potential of the reinforcement reach lower values: E_st,5_ = −300 mV and E_st,17_ = −267 mV, indicating a 50% probability of corrosion. 

Meanwhile, in the specimens where the air entrained was not added, the half-cell potential of the reinforcement reached the values of E_st_ ≈ −300 mV and lower, even reaching E_st,2_ = −521 mV (Figure 10), which indicated a 95% probability of reinforcement corrosion. 

### 3.3. The Concrete Cover Resistivity

**Figure 11:**
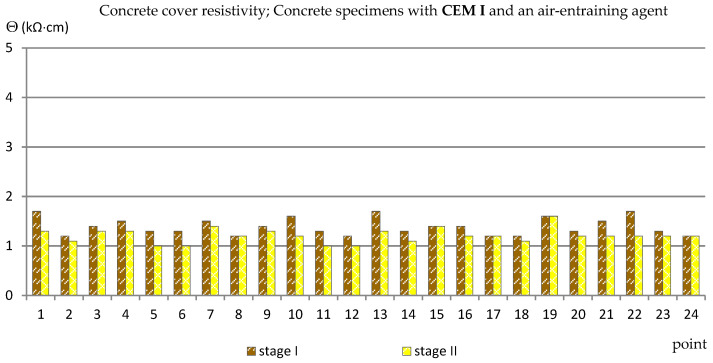
Concrete cover resistivity measured in C_I_a specimens.

**Figure 12:**
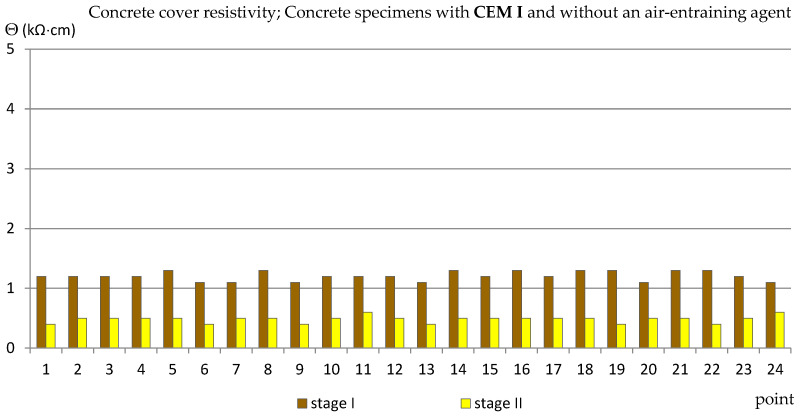
Concrete cover resistivity measured in C_I_n specimens.

**Figure 13:**
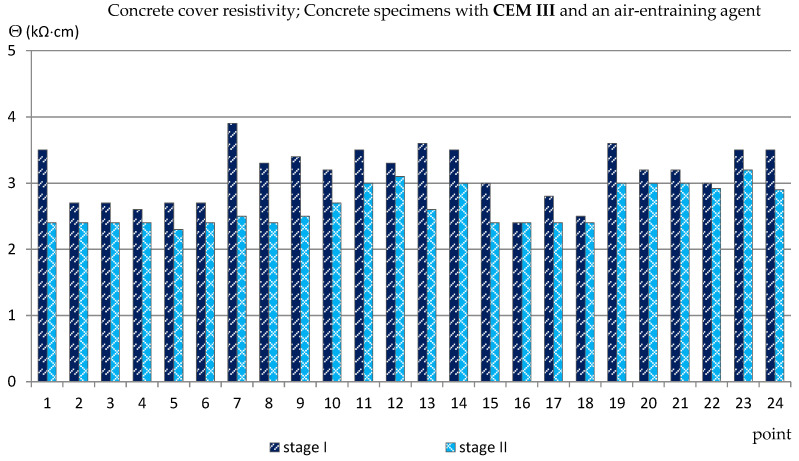
Concrete cover resistivity measured in C_III_a specimens.

**Figure 14:**
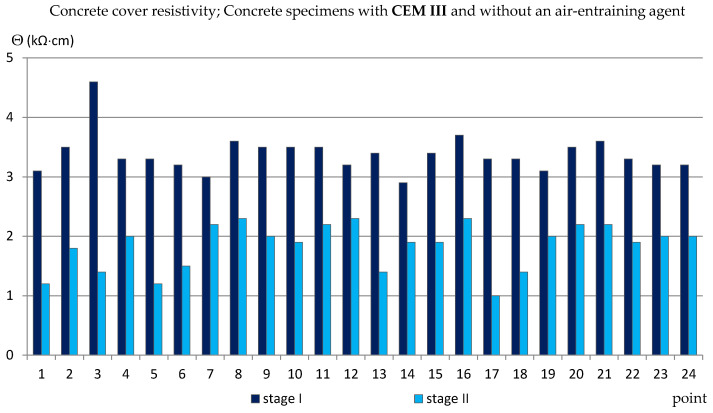
Concrete cover resistivity measured in C_III_n specimens.

Similarly to the measurements of the half-cell potential of reinforcement, also the measurements of the concrete cover resistivity provided only estimated results allowing us to determine the probability of corrosion of the reinforcement in the studied area. In addition, the analysis of the results of measurements of the concrete cover resistivity in this case gave quite surprising results in relation to the criteria in Table 2. The results obtained from the reference measurements, regardless of the type of specimens tested, at each of the measurement points were lower than Θ = 10 (kΩ·cm), indicating a high probability of corrosion, which was rather impossible. Such results have already appeared in the author’s research [35,39,48], always during measurements performed on relatively young specimens. This was probably due to the fact that concrete is a non-stabilized material in which physico-chemical processes take place long after the mixture has set (even for years) and have an impact on the internal structure of concrete [2,4]. Moreover, the time-dependent change of concrete resistivity recomputed to a diffusion coefficient of chlorides into concrete was observed in a set of SCM-based concrete mixtures [49]. Nevertheless, the differences between the values obtained from the reference measurements and measurements taken after 120 cycles of freezing and thawing specimens in a 3% NaCl solution allowed for capturing the trend of changes depending on the cement and the air-entraining agent used (Figure 11, Figure 12, Figure 13 and Figure 14, Table 5). In the case of the reinforcement concrete cover analysis, the higher its value, the more effective the concrete cover is as a layer protecting the reinforcement against corrosion. Table 5 shows the extreme values of the concrete cover resistivity obtained from measurements of all types of specimens before and after the cycles of freezing and thawing samples in an NaCl solution.

In specimens with Portland cement, in which an air-entraining agent was used (C_I_a), the values of the concrete cover resistivity obtained from the reference measurements were Θ = 1.2 ÷ 1.7 (kΩ·cm) and decreased slightly after the freezing cycles, to the value of Θ = 1.0 ÷ 1.6 (kΩ·cm) (Figure 11).

However, in the specimens in which no air-entraining agent was used (C_I_n), the decrease in the value of the concrete cover resistivity was much greater: from: od Θ ≈ 1.2 (kΩ·cm) (reference measurements) to Θ ≈ 0.5 (kΩ·cm) (measurements after freezing cycles) as shown in Figure 12. 

The values of the concrete cover resistivity obtained from the measurements made on the specimens with blast-furnace slag cement were higher than for the samples with Portland cement. Reference measurements made on C_III_a specimens gave the values of Θ = 2.4 ÷ 3.9 (kΩ·cm) and fell after the freezing cycles to the value of Θ = 2.3 ÷ 3.2 (kΩ·cm) (Figure 13). 

On the other hand, in the specimens without an aerating agent (C_III_n), the difference between the reference values and those obtained after the freezing cycles was much greater, on average from Θ ≈ 3.5 (kΩ·cm) to Θ ≈ 1.8 (kΩ·cm) (Figure 14). 

### 3.4. Microstructural Studies Using a Scanning Electron Microscope (SEM Analysis)

**Figure 15:**
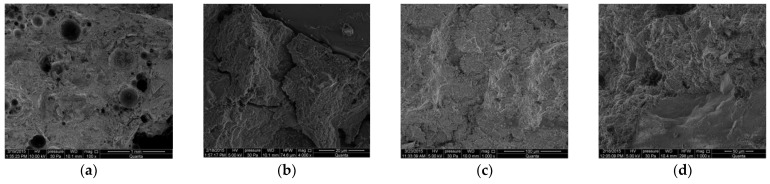
Photos of the microstructure of concrete taken from the cover layer of the tested samples: (**a**) C_I_a, (**b**) C_I_n, (**c**) C_III_a, (**d**) C_III_n.

Additionally, using a scanning electron microscope (SEM), the microstructure of concrete collected from the cover layer was observed for randomly selected samples from each group: C_I_a, C_I_n, C_III_a, C_III_n. In Figure 15, there are exemplary photos obtained as a result of the observation. Unfortunately, different magnification rates make it difficult to accurately compare the structure of concrete in samples of different types. Nevertheless, it is noticeable that the concrete of sample C_III_a (i.e., the concrete with blast-furnace slag cement and air-entraining agent, shown in Figure 15c) has the most compact structure. On the other hand, the greatest number of microcracks is visible in C_I_n concrete (i.e., concrete with Portland cement without air-entraining agent, Figure 15b). It follows that the damage to the microstructure caused by cyclic freezing and thawing is much smaller in air-entrained concretes, because the dispersed air bubbles take up the internal stresses caused by the freezing of the liquid in the pores, which reduces the damage. As a result, the possibility of chloride ions penetrating into the cover is limited—the capillary absorption coefficient of chlorides in air-entrained concretes is lower than in concretes in which no air-entraining agent was used. Thus, the corrosion of the reinforcement is limited [50].

## 4. Conclusions

The paper presents tests that allow us to determine what type of cement (Portland cement or blast-furnace slag cement) and the addition of an air-entraining agent most effectively affect the properties of the concrete cover as a layer protecting the reinforcing bars against corrosion caused by the simultaneous action of chloride ions and frost.

The assessment of the probability of corrosion in the tested specimens and the corrosion activity of the reinforcement were tested using the galvanostatic pulse technique. The use of this method made it possible to measure three parameters in a relatively uncomplicated and quick way, i.e., corrosion current density, half-cell potential of the reinforcement and resistivity of the concrete cover and the obtained values allowed for a comparative analysis of the tested specimens.

1. Based on the results obtained from the measurements of the corrosion current density (the most reliable parameter), it was found that after the specimens were subjected to 120 freezing and thawing cycles in a 3% NaCl solution, the lowest corrosion activity of the reinforcement was recorded on the reinforcement in C_III_a specimens, i.e., specimens made of concrete with blast-furnace slag cement and an air-entraining agent addition. On the other hand, the use of blast-furnace slag cement for concrete, but without adding an air-entraining agent (C_III_n specimens), although this cement should protect against chloride corrosion, does not reduce the corrosive activity of the reinforcement. The results obtained on C_III_n specimens are comparable to the results obtained on C_I_a and C_I_n specimens. On the other hand, concrete with the addition of an air-entraining agent in which Portland cement was used (C_I_a specimens) is not effective in protecting against the simultaneous effects of chloride corrosion and frost.
2. The results obtained from the measurements of the reinforcement half-cell potential and the concrete cover resistivity were less reliable than the measurement of the corrosion current density and m might be influence by concrete mixture and age of evaluation. Nevertheless, they confirmed the previous analyses. Concrete in C_III_a specimens turned out to be more effective in reducing the probability of reinforcement corrosion.
3. Moreover, it was found that the measurements of the concrete cover resistivity performed by the galvanostatic pulse method are not very reliable in the case of tests on young (several months old) specimens.
4. The observation of the microstructure of concrete under a scanning electron microscope showed that the most compact structure is characterized by concrete with blast-furnace slag cement and the addition of an air-entraining agent taken from the C_III_a specimens.

## Figures and Tables

**Table 1:** Components of the concrete mix (kg/m^3^).

Group of Specimen	CEM I 42.5 N-MSR/NA	CEM III/A 42, 5 N-LH/HSR/NA	Basalt Grit f 2/8 mm	Basalt Grit f 8/16 mm	Mine Sand	Water	Plasticizer—Adva Flow 440	Air-Entraining Agent—Darex AEA W (LP)
C_I_a	384	—	600	650	680	166	0.6%	0.5%
C_I_n								—
C_III_a	—	384						0.5%
C_III_n								—

**Table 2:** Criteria for assessing the degree of reinforcement corrosion risk.

		Criteria for Assessing the Degree of Reinforcement Corrosion Risk ^1^		
Advanced measurements		Corrosion current density, i_cor_ (μA/cm^2^)	<0.5	not forecasted corrosion activity
			0.5 ÷ 2.0	irrelevant corrosion activity
			2.0 ÷ 5.0	low corrosion activity
			5.0 ÷ 15.0	moderate corrosion activity
	Basic measurements	Reinforcement half-cell potential, E_st_ (mV)	>−200	5% of corrosion probability
			−350 ÷ −200	50% of corrosion probability
			<−350	95% of corrosion probability
		Concrete cover resistivity, Θ (kΩ·cm)	≥20	small corrosion probability
			10 ÷ 20	medium corrosion probability
			≤10	high corrosion probability

^1^ The criteria in this Table are appropriate for measurements made with the GP-5000 GalvaPulse^TM^ instrument. It is not appropriate to compare reference values with measurements obtained with other devices [26].

**Table 3:** Minimum and maximum values of corrosion current density obtained from measurements before and after cycles of freezing and thawing specimens in a 3% NaCl solution.

Corrosion Current Density, i_cor_ (μA/cm^2^)	CEM_I_a		CEM_I_n		CEM_III_a		CEM_III_n	
	Min	Max	Min	Max	Min	Max	Min	Max
Reference measurement	0.56	1.48	0.84	1.45	0.72	1.44	0.91	3.07
Measurement after 120 cycles of freezing and thawing in 3% NaCl solution	2.59	10.19	2.82	9.87	0.82	3.86	2.51	9.72

**Table 4:** Minimum and maximum values of reinforcement half-cell potential obtained from measurements before and after cycles of freezing and thawing specimens in a 3% NaCl solution.

Reinforcement Half-Cell Potential, E_st_ (mV)	CEM_I_a		CEM_I_n		CEM_III_a		CEM_III_n	
	Min	Max	Min	Max	Min	Max	Min	Max
Reference measurement	−132	−257	−6	−149	−91	−189	−68	−284
Measurement after 120 cycles of freezing and thawing in 3% NaCl solution	−189	−341	−290	−378	−129	−300	−275	−521

**Table 5:** Minimum and maximum values of concrete cover resistivity obtained from measurements before and after cycles of freezing and thawing specimens in a 3% NaCl solution.

Concrete Cover Resistivity, Θ (kΩ·cm)	CEM_I_a		CEM_I_n		CEM_III_a		CEM_III_n	
	Min	Max	Min	Max	Min	Max	Min	Max
Reference measurement	1.2	1.7	1.1	1.3	2.4	3.9	2.9	4.6
Measurement after 120 cycles of freezing and thawing in 3% NaCl solution	1.0	1.6	0.4	0.6	2.3	3.2	1.0	3.0

## Data Availability

The data presented in this study are available on request from the corresponding author.

## References

[1] Green W., Chess P. (2019). Durability of Reinforced Concrete Structures.

[2] Ściślewski Z. (1999). Durability of Reinforced Concrete Structures.

[3] Bertolini L., Elsener B., Pedeferri P., Polder R. (2004). Corrosion of Steel in Concrete.

[4] Kurdowski W. (2014). Cement and Concrete Chemistry.

[5] Verma S.K., Bhadauria S.S., Akhtar S. (2014). Monitoring corrosion of steel bars in reinforced concrete structures. Sci. World J..

[6] Yeomans S.R. (2016). Galvanized steel reinforcement. Corrosion of Steel in Concrete Structures.

[7] Jaśniok M., Kołodziej J., Gromysz K. (2021). An 18-month analysis of bond strength of hot-dip galvanized reinforcing steel B500SP and S235JR+AR to chloride contaminated concrete. Materials.

[8] Jaśniok M., Sozańska M., Kołodziej J., Chmiela B. (2020). A two-year evaluation of corrosion-induced damage to hot galvanized reinforcing steel b500sp in chloride contaminated concrete. Materials.

[9] Manalo A., Maranan G., Benmokrane B., Cousin P., Alajarmeh O., Ferdous W., Liang R., Hota G. (2020). Comparative durability of GFRP composite reinforcing bars in concrete and in simulated concrete environments. Cem. Concr. Compos..

[10] Khotbehsara M.M., Manalo A., Aravinthan T., Ferdous W., Nguyen K.T., Hota G. (2020). Ageing of particulate-filled epoxy resin under hygrothermal conditions. Constr. Build. Mater..

[11] Thamrin R. (2016). Effect of end anchorage length and stirrup ratio on bond and shear capacity of concrete beams with nonmetallic reinforcement. J. Eng. Sci. Technol..

[12] Babiak I. (2021). Research of non-metal composite basalt reinforcement of periodic profile and prospects of its use. Dorogi Mosti.

[13] Mosley C.P., Tureyen A.K., Frosch R.J. (2008). Bond strength of nonmetallic reinforcing bars. Aci. Struct. J..

[14] Ekenel M., y Basalo F.D.C., Nanni A. (2021). Fiber-Reinforced Polymer Reinforcement for Concrete Members, ACI Committee 440 is Taking the Next Step toward Building Code Compliance. www.concreteinternational.com.

[15] Raczkiewicz W. (2016). Effect of concrete addition of selected micro-fibers on the reinforcing bars corrosion in the reinforced concrete specimens. Adv. Mater. Sci..

[16] Ye H., Jin N. (2019). Degradation mechanisms of concrete subjected to combined environmental and mechanical actions: A review and perspective. Comput. Concr..

[17] Czarnecki L., Emmons P.H. (2002). Repair and Protection of Concrete Structures.

[18] Luo D., Li Y., Li J., Lim K.-S., Nazal N.A.M., Ahmad H. (2018). A recent progress of steel bar corrosion diagnostic techniques in rc structures. Sensors.

[19] Jaśniok M., Jaśniok T. (2017). Measurements on corrosion rate of reinforcing steel under various environmental conditions, using an insulator to delimit the polarized area. Procedia Eng..

[20] CEN (2004). PN-EN 1992-1-1:2008 Eurocode 2: Design of Concrete Structures—Part 1-1: General Rules and Rules for Buildings.

[21] Owsiak Z., Grzmil W. (2014). The evaluation of the influence of mineral additives on the durability of self-compacting concretes. KSCE J. Civ. Eng..

[22] Raczkiewicz W., Grzmil W. (2017). Assessment of the impact of cement type on the process of concrete carbonation and reinforcement corrosion in reinforced concrete specimens. Cem. Lime Concr..

[23] Aitcin J.C. (1998). High-Performance Concrete.

[24] Małolepszy J. Durability of concretes made of slag cements. Proceedings of the Scientific-Technical Conference.

[25] Giergiczny Z. (2010). Cements with mineral additives as a component of durable concrete. Eng. Constr..

[26] Deja J. (2007). Corrosion durability of binders with different content of granulated blast furnace slag. Cement. Lime. Concr..

[27] Liu J., Jiang Z., Zhao Y., Zhou H., Wang X., Zhou H., Xing F., Li S., Zhu J., Liu W. (2020). Chloride distribution and steel corrosion in a concrete bridge after long-term exposure to natural marine environment. Materials.

[28] Wang Y., Liu C., Li Q., Wu L. (2019). Chloride ion concentration distribution characteristics within concrete covering-layer considering the reinforcement bar presence. Ocean Eng..

[29] Kuziak J., Woyciechowski P.P., Kobyłka R., Wcisło A. (2018). The content of chlorides in blast-furnace slag cement as a factor affecting the diffusion of chloride ions in concrete. MATEC Web Conf..

[30] Coppola L., Coffetti D., Crotti E., Gazzaniga G., Pastore T. (2020). Chloride Diffusion in Concrete Protected with a Silane-Based Corrosion Inhibitor. Materials.

[31] Hájková K., Šmilauer V., Jendele L., Červenka J. (2018). Prediction of reinforcement corrosion due to chloride ingress and its effects on serviceability. Eng. Struct..

[32] Rusin Z. (2002). Technology of Frost-Resistant Concrete.

[33] Czarnecki L., Deja J., Flaga K., Kurdowski W., Małolepszy J., Radomski W., Śliwiński J. (2015). Concrete frost resistance in bridge structures. Constr. Technol. Archit..

[34] Wawrzeńczyk J., Molendowska A., Juszczak T. (2018). Determining k-value with regard to freeze-thaw resistance of concretes containing GGBS. Materials.

[35] Raczkiewicz W. (2018). Influence of the air-entraining agent in the concrete coating on the reinforcement corrosion process in case of simultaneous action of chlorides and frost. Adv. Mater. Sci..

[36] GalvaPulse. http://www.germann.org/TestSystems/GalvaPulse/GalvaPulse.pdf.

[37] Helal J., Sofi M., Mendis P. (2015). Non-destructive testing of concrete: A review of methods. Electron. J. Struct. Eng..

[38] Hoła J., Bien J., Sadowski L., Schabowicz K. (2015). Non-destructive and semi-destructive diagnostics of concrete structures in assessment of their durability. Bull. Pol. Acad. Sci. Tech. Sci..

[39] Raczkiewicz W. (2019). Building Diagnostics. Selected Methods of Materials as Well as Elements and Structures Test.

[40] Klinghoffer O. (1995). In situ monitoring of reinforcement corrosion by means of electrochemical methods. Nord. Concr. Res..

[41] Elsner B., Klinghoffer O., Frolund T., Rislund E., Schiegg Y., Böhni H. Assessment of reinforcement corrosion by means of galvanostatic pulse technique. Proceedings of the International Conference Repair of Concrete Structures.

[42] Frølund T., Klinghoffer O., Poulsen E. Rebar Corrosion Rate Measurements for Service Life Estimates. Proceedings of the ACI Fall Convention.

[43] Vedalakshmi R., Balamurugan L., Saraswathy V., Kim S.-H., Ann K.Y. (2010). Reliability of galvanostatic pulse technique in assessing the corrosion rate of rebar in concrete structures: Laboratory vs. field studies. KSCE J. Civ. Eng..

[44] ASTM (2009). Standard test method for half-cell potentials of uncoated reinforcing steel in concrete. American Society of Testing and Materials.

[45] Raczkiewicz W., Wójcicki A. (2017). Some aspects of the reinforcing steel corrosion level prediction in concrete using electrochemical method. Weld. Technol. Rev..

[46] Tworzewski P., Raczkiewicz W., Czapik P., Tworzewska J. (2021). Diagnostics of concrete and steel in elements of an historic reinforced concrete structure. Materials.

[47] Ghosh P., Tran Q. (2014). Correlation between bulk and surface resistivity of concrete. Int. J. Concr. Struct. Mater..

[48] Raczkiewicz W., Kossakowski P.G. (2019). Electrochemical diagnostics of sprayed fiber-reinforced concrete corrosion. Appl. Sci..

[49] Tran Q., Ghosh P., Lehner P., Konečný P. (2020). Determination of time dependent diffusion coefficient aging factor of HPC mixtures. Key Eng. Mater..

[50] Zhu F., Ma Z., Zhao T. (2016). Influence of freeze-thaw damage on the steel corrosion and bond-slip behavior in the reinforced concrete. Adv. Mater. Sci. Eng..

